# Cross-Species Antiviral Activity of Goose Interferons against Duck Plague Virus Is Related to Its Positive Self-Feedback Regulation and Subsequent Interferon Stimulated Genes Induction

**DOI:** 10.3390/v8070195

**Published:** 2016-07-18

**Authors:** Hao Zhou, Shun Chen, Qin Zhou, Yunan Wei, Mingshu Wang, Renyong Jia, Dekang Zhu, Mafeng Liu, Fei Liu, Qiao Yang, Ying Wu, Kunfeng Sun, Xiaoyue Chen, Anchun Cheng

**Affiliations:** 1 Institute of Preventive Veterinary Medicine, Sichuan Agricultural University, No. 211 Huimin Road, Wenjiang District, Chengdu 611130, China; zhouhao19911030@163.com (H.Z.); 18980768983@163.com (Q.Z.); 15183523348@163.com (Y.W.); mshwang@163.com (M.W.); cqrc_jry@163.com (R.J.); liumafengra@163.com (M.L.); yangqiao721521@sina.com (Q.Y.); yingzi_no1@126.com (Y.W.); sunkunfeng1981@163.com (K.S.); 2 Avian Disease Research Center, College of Veterinary Medicine, Sichuan Agricultural University, Chengdu 611130, China; zdk24@sicau.edu.cn (D.Z.); chenxy_24@sina.cn (X.C.); 3 Key Laboratory of Animal Disease and Human Health of Sichuan Province, Sichuan Agricultural University, Chengdu 611130, China; liufei@sicau.edu.cn

**Keywords:** goose interferon, duck plague virus, feedback regulation, antiviral activity, interferon stimulated gene

## Abstract

Interferons are a group of antiviral cytokines acting as the first line of defense in the antiviral immunity. Here, we describe the antiviral activity of goose type I interferon (IFNα) and type II interferon (IFNγ) against duck plague virus (DPV). Recombinant goose IFNα and IFNγ proteins of approximately 20 kDa and 18 kDa, respectively, were expressed. Following DPV-enhanced green fluorescent protein (EGFP) infection of duck embryo fibroblast cells (DEFs) with IFNα and IFNγ pre-treatment, the number of viral gene copies decreased more than 100-fold, with viral titers dropping approximately 100-fold. Compared to the control, DPV-EGFP cell positivity was decreased by goose IFNα and IFNγ at 36 hpi (3.89%; 0.79%) and 48 hpi (17.05%; 5.58%). In accordance with interferon-stimulated genes being the “workhorse” of IFN activity, the expression of duck myxovirus resistance (Mx) and oligoadenylate synthetases-like (OASL) was significantly upregulated (*p* < 0.001) by IFN treatment for 24 h. Interestingly, duck cells and goose cells showed a similar trend of increased ISG expression after goose IFNα and IFNγ pretreatment. Another interesting observation is that the positive feedback regulation of type I IFN and type II IFN by goose IFNα and IFNγ was confirmed in waterfowl for the first time. These results suggest that the antiviral activities of goose IFNα and IFNγ can likely be attributed to the potency with which downstream genes are induced by interferon. These findings will contribute to our understanding of the functional significance of the interferon antiviral system in aquatic birds and to the development of interferon-based prophylactic and therapeutic approaches against viral disease.

## 1 Introduction

Pattern recognition receptors (PRRs) senses foreign agents in response to pathogen-associated molecular patterns (PAMPs), inducing the production of interferons and proinflammatory cytokines. Interferons, secreted antiviral cytokines that induce a robust immune response, play an important role in both innate and adaptive immunity [1]. IFNs are classified into three classes based on the receptor complex through which signaling occurs: type I interferon (e.g., IFNα), type II interferon (IFNγ), and type III interferon (IFNλ1, 2, 3) [2]. The functions of interferons have recently been identified in several avian species. Chicken IFNα can suppress the replication of several viruses, such as infectious bronchitis virus (IBV) [3], infectious bursal disease virus (IBDV) [4], and Marek’s disease virus (MDV) [5]. Pretreatment of Vero cells with chicken IFNγ effectively inhibits vesicular stomatitis virus (VSV) infection [6]. In addition, duck IFNα [7] and IFNγ [8] exhibit a strong inhibitory effect against duck hepatitis B virus (DHBV) in primary duck hepatocytes. Recombinant goose IFNα produced by either *E. coli* or Sf9 has been shown to be a powerful antiviral agent [9]. Goose IFNγ has an antiviral effect against goose paramyxovirus (GPMV) in goose fibroblasts and inhibits vesicular stomatitis virus expressing enhanced green fluorescent protein (VSV-EGFP) replication in duck fibroblasts [10]. Previous research has also demonstrated the cross-species reactivity of turkey and chicken interferons [11,12]. How is the host cellular antiviral state achieved? Interferons bind to their cognate receptors, inducing the expression of interferons and a variety of IFN-stimulated genes (ISGs) [13] such as myxovirus resistance (Mx) protein [14], oligoadenylate synthetases-like (OASL) protein [15], and dsRNA-dependent protein kinase (PKR) [16] and resulting in the antiviral response [2]. Importantly, a positive feedback loop for interferon through autocrine and paracrine pathways via distinct IRF proteins (e.g., IRF3 and IRF7) has been extensively studied in mammals [17,18] and shown to further massively amplify responses by interferon and related ISGs. However, the mechanism of positive feedback regulation of IFNs in birds remains unclear. Considering that aquatic birds play a critical role in the transmission and dissemination of many important viral pathogens, it is important to study the IFN-mediated antiviral immunity in waterfowl. Duck and goose showed a recent phylogenetic relationship. The goose IFNα and duck IFNα is 93.7% [9], while the goose IFNγ and duck IFNγ is the 93.3% [10]. Furthermore, limited attention has been paid to the antiviral response of goose IFN proteins. Notably, most of these antiviral proteins, including Mx and OASL, are not well described in the geese, and their roles against viral infections is unknown. Duck plague virus (DPV) (also known as the etiological agent of duck virus enteritis), is a DNA virus detected in many species, including ducks, geese, swans, and other waterfowls, which leads to the obvious economic losses worldwide in avian industry as a result of high mortality [19,20,21,22]. Migratory aquatic birds (goose) and domestic aquatic birds (duck) may spread the virus infection from one species to another.

In the present study, we examined the potential antiviral activity and explored some novel immune regulatory characteristics of goose IFNα (goIFNα) and IFNγ (goIFNγ) against DPV in duck embryo fibroblast cells. We observed significant inhibition of DPV replication by both goIFNα and goIFNγ in vitro. We then focused on an analysis of host ISG expression and viral replication during the infection phase. Here, evidence was obtained for the antiviral effect of goose interferon on the heterologous duck cells. The primary investigation of the cross-species antiviral activity of goose IFNs in duck-derived cells indicated that goose IFNs can be exploited into a library of small antiviral molecules that can be used in multiple animal viral disease treatment. The ultimate goal of the work is to develop a multi-function and multi-target antiviral reagents.

## 2. Materials and Methods

### 2.1. Cells and Virus

Baby Hamster Syrian Kidney (BHK21) cells were provided by our lab. Duck plague virus strain (DPV-EGFP) was constructed and stored at −80 °C until use. Unless otherwise stated, the virus tissue culture infectious dose 50 (TCID_50_) in duck embryo fibroblast cells used was 10^−6.125^/100 µL. Virus was seeded into 6-well (4 × 10^4^ TCID_50_) or 24-well plates (10^4^ TCID_50_). 1 × 10^6^ primary duck embryo fibroblast cells (DEF) or goose embryo fibroblast cells (GEF) were seeded into 6-well tissue culture plates for 24 h in DMEM (Sigma, St. Louis, MO, USA) supplemented with 10% fetal bovine serum (FBS) 10%, *v/v*, (Gibco, Gaithersburg, MD, USA), penicillin 100 IU/mL and streptomycin 100 μg/mL. The cells were then treated with protein IFNα and IFNγ separately for 12 h. The DEFs were then inoculated with DPV-EGFP in DMEM supplemented with 3% FBS. Viral DNA was extracted using TIANamp Virus DNA/RNAKit (Tiangen, Beijing, China), and RT-qPCR was performed.

### 2.2. Plasmid Construction and Transfection

The goIFNα (GenBank No. EU925650) and the goIFNγ (GenBank No. KP325480) sequences tagged with His at the N-terminus but without the secretion signal peptide coding region were cloned into a modified pcDNA3.1 (+) vector to generate the pcDNA3.1-IFNα-His plasmids. BHK21 cells were transiently transfected in T25 with 7 μg IFN recombinant plasmid using 21 μl of TransIT-X2 transfection reagent (Mirus Bio, Madison, WI, USA) according to the manufacturer’s guidelines. At 24 h after transfection, supernatant of the BHK21 cell lysates were collected and stored at −80 °C until further use. The control group is the supernatant collected from the BHK21 cells transfected with the empty pcDNA3.1 plasmid. The goose IFNα and IFNγ sequences containing the secretion signal peptide region were also inserted into the pEGFP-N1 vector using a one-step cloning kit (Vazyme, Nanjing, China) to generate the recombinant pEGFP-IFNα and pEGFP-IFNγ plasmids, respectively. Transfection into the BKH21 cells in 6-well plates (2 µg plasmid/well) was performed according to the manufacturer’s guidelines. The expression levels of goIFNα and goIFNγ were confirmed by Western Blotting. The primers are listed in Table 1.

### 2.3. Viral TCID_50_ Detection

Viral titers were determined by an endpoint dilution assay and the titers are expressed as the TCID_50_ per milliliter using the Reed-Muench method. Briefly, serial 10-fold dilutions of DPV-EGFP were inoculated in eight replicates into 96-well tissue culture plates seeded with DEF cells. After absorption for 1 h at 37 °C, the supernatants in the wells were removed, and DMEM with 3% FBS was added. The plates were incubated for 120 h, and the virus titers were calculated based on the cytopathic effect.

### 2.4. RNA Isolation and Real-Time qPCR

Total RNA was isolated from the DEFs or GEFs using TRIzol Reagent (Takara, Dalian, China) following the manufacturer’s instructions. cDNA was synthesized from 1 µg of RNA per sample using 5× all-in-one master mix transcription reagents (Abm, Richmond, BC, Canada). Relative expression was then quantified using the SYBR Green qPCR kit (Abm, Richmond, BC, Canada) and a real-time Thermo cycler (CFX96 Bio-Rad, Hercules, CA, USA). qPCR was performed using primers (Table 1) for duck genes (duIFNα, duIFNγ, duIFNλ, duMx, and duOASL) and goose genes (goIFNα, goIFNγ, goIFNλ, goMx, and goOASL). The relative expression of the target genes was normalized to β-actin and calculated using the 2^−ΔΔCT^ method [23].

### 2.5. Flow Cytometry Analysis

DEFs were grown in 6 wells plates or 24-well plates for 12 h. Then, the DEF monolayers were pretreated with the indicated IFN protein (60 µg/well) and negative control (supernatants of cellular lysate from BHK21 transfected with empty vector); 12 h later, the cells were then infected by DPV-EGFP (4 × 10^4^ TCID_50_/well). DPV-infected cells at the indicated time points (36 hpi and 48 hpi) were detached from the plate bottom and then washed with PBS. Then, these cells were harvested by using the trypsin and centrifuged at 1000 rpm for 5 min before being washed twice with PBS again and finally suspended in 0.5 mL PBS. The percentage of GFP-positive cells was determined by flow cytometry (Becton Dickinson, San Jose, CA, USA). Uninfected cells were used as a negative control.

### 2.6. Detection of Viral Copies

An absolute quantitative curve was built based on the DPV-UL30 plasmid. The plasmid was diluted 10-fold. Then, the temperature was optimized by the program and the standard curve of the DPV was generated from the 10^8^ copies to 10^4^ copies. Then, the viral DNA from the infected cells was extracted using a nucleic acid extraction kit (Tiangen, Beijing, China), and the samples were detected by the following program: 94 °C for 3 min, followed by 39 cycles of 95 °C for 10 s and 60 °C for 30 s. The primers are listed in Table 1, and the targeted product was 106 bp.

### 2.7. Western Blotting

The protein concentrations were calculated using the Bradford assay (Bio-Rad). Whole cell lysates of BHK21 cells were collected at 24 h or 48 h post-transfection by three rounds of freeze-thaw. Unless otherwise stated, a total of 20 µg of the total cellular protein was boiled in 6× protein loading buffer before separation by 12% SDS-PAGE electrophoresis. Then, the proteins were transferred onto polyvinylidene fluoride (PVDF) membranes (Millipore, Bedford, MA, USA). The membranes were blocked with 5% skim milk in TBST overnight at 4 °C, and subsequently incubated for 1 h with mouse anti-His (Proteintech, Shenzhen, China), Rabbit anti-actin monoclonal antibodies (Bioss, Beijing, China) or mouse anti-GAPDH monoclonal (Ruiying Biological, Suzhou, China) antibodies at a 1:2000 dilution. Horseradish peroxidase (HRP)-conjugated goat anti-rabbit or goat anti-mouse IgG (Earthox, San Francisco, CA, USA) was used as the secondary antibody at 1:5000 dilution. Proteins were visualized by chemiluminescence using an ECL kit (Bio-Rad).

### 2.8. Statistical Analysis

The statistical significance of differences between experimental groups was determined by a two-tailed unpaired Student’s *t*-test (GraphPad Prism software). Error bars represent the standard error of the mean. The value *p* < 0.05 was considered statistically significant, and the degree of significance is indicated as follows: * *p* < 0.05, ** *p* < 0.01, *** *p* < 0.001.

## 3. Results

### 3.1. Characterization of Goose IFNα and IFNγ Expression

**Figure 1:**
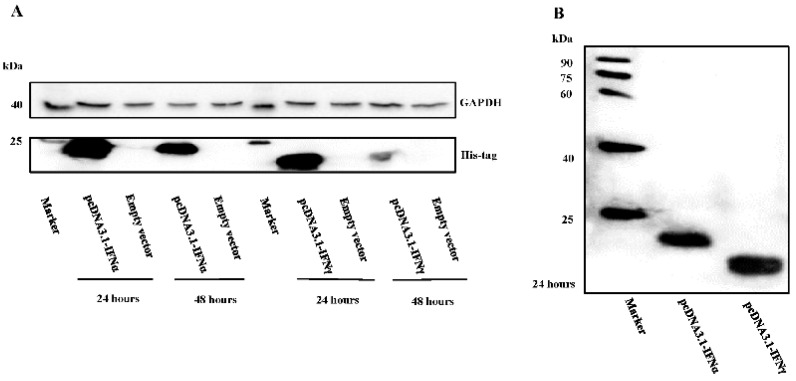
Western blot analysis of goose type I interferon (IFNα) and type II interferon (IFNγ) expression in Baby Hamster Syrian Kidney (BHK21) cells. (**A**) BHK21 cells were transfected with the empty vector or a pcDNA3.1-vector expressing goose IFNα or IFNγ. Cell lysates after transfection for 24 h and 48 h, were examined by Western Blotting with anti-His tag antibodies and anti-GAPDH antibodies as a loading control; (**B**) Western Blotting analysis (24 hpi) showed approximate sizes for recombinant goose IFNα and IFNγ of 20 kDa and 18 kDa, respectively.

The pcDNA3.1-IFNα and pcDNA3.1-IFNγ plasmids were successfully constructed. After transfection into the BHK21 cells, expression at 24 h and 48 h post-transfection was observed for both, with a higher expression level of goose IFNs observed at 24 h post transfection (Figure 1A). To explore the goose IFN expression pattern in BHK21 cells, their subcellular localization was assessed. Western blot analysis showed that the recombinant goIFNα and goIFNγ proteins are approximately 20 kDa and 18 kDa, with an approximately 1 kDa His tag, respectively (Figure 1B). Fluorescence was observed for the the goose IFNα-EGFP and IFNγ-EGFP proteins (Figure S1). Morphologies of IFNα appeared in the dots-, strings- and rings-like patterns diffusely distributed along the periphery of the nucleus. Morphologies of IFNγ showed patterns of dots, spots, stars and cocoons, located in the cell nucleus and perinuclear compartment. Taking the results together, the overexpression of goose IFN proteins in BHK21 cells was confirmed.

### 3.2. Antiviral Effect of Goose IFNα and IFNγ

**Figure 2:**
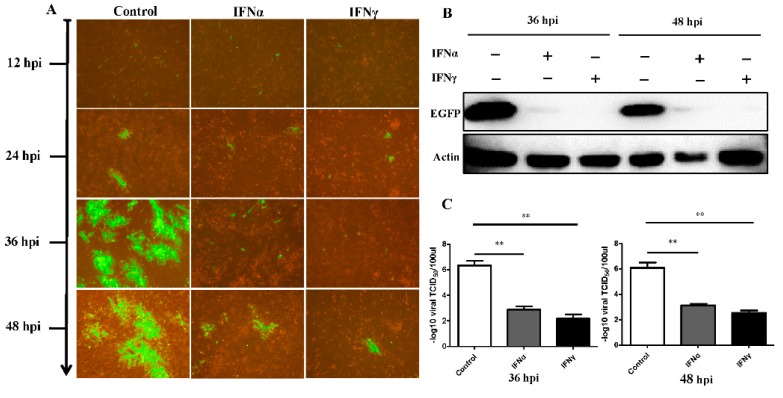
Duck plague virus (DPV) was significantly inhibited by the goose IFNα and IFNγ. (**A**) duck embryo fibroblast (DEF) cells were pre-treated with indicated interferon protein and negative control (60 µg/well). The the supernatant collected from the BHK21 cells transfected with the empty pcDNA3.1 (+) plasmids is used to pre-treat GEFs as the negative control. 12 h later, the cells were then infected by DPV-EGFP (4 × 10^4^ TCID_50_/well). Enhanced green fluorescent protein (EGFP) expression, an indication of DPV replication, is shown by green fluorescence when examined by fluorescence microscopy. Magnification 400×; (**B**) At 36 hpi and 48 hpi, the medium was collected for the further Western Blotting analysis. The primary antibodies were rabbit-anti EGFP (1:2000) and mouse-anti actin (1:2000); secondary antibodies were the goat-anti rabbit and goat-anti mouse antibodies conjugated to Horseradish peroxidase (HRP); (**C**) Viral titer reduction assay. Cells were treated with indicated IFNα, IFNγ, or left control for 12 h before infection with DPV. After 36 h and 48 h, the samples in the four wells used for each treatment were frozen and thawed out repeatedly and pooled, and the viral yield in the culture medium was determined by the tissue culture infectious dose 50 (TCID 50) method.

**Figure 3:**
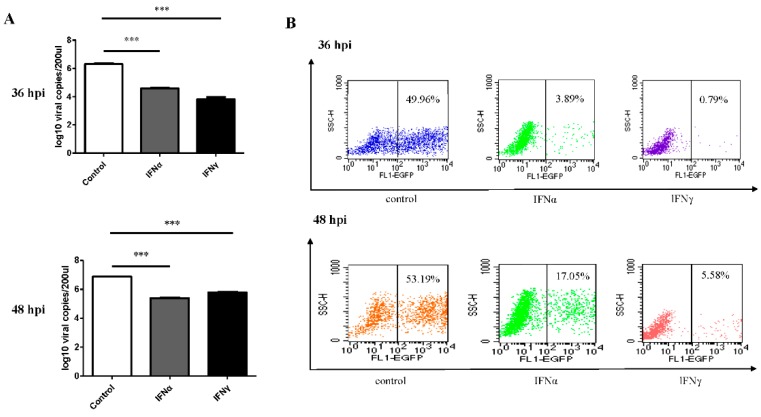
Effects of IFNα and IFNγ on DPV DNA copy numbers and viral replication. (**A**) DPV UL30 DNA copies were detected by RT-qPCR in DEF cells infected with the DPV strain at 36 hpi and 48 hpi after pretreatment of goose proteins (60 µg/well) from BHK21 cells transfected with the empty (negative control), or IFNα or IFNγ plasmid. The numbers of DPV UL30 gene copies was were calculated according to the standard curve. The assays were performed in triplicate wells, *** *p* < 0.001; (**B**) Flow cytometry analysis of DPV-EGFP positive cells from corresponding samples at 36 hpi and 48 hpi.

To explore the antiviral effect, a DPV infection model was generated. The results showed cross-specificity for goose interferons in viral inhibition. Specifically, the DPV seeded into DEFs replicated after 24 hpi. At 12 hpi and 24 hpi, there were no obvious differences between the IFN-treatment group and control group. However, dramatic antiviral effects were observed at both 36 hpi and 48 hpi (Figure 2A). Western blot analysis revealed that IFNα and IFNγ controlled the DPV-EGFP expression and proliferation (Figure 2B). Furthermore, the virus titers in the presence of the interferons rapidly decreased to low levels (10^2.875^ TCID_50_/0.1 mL in the IFNα group and 10^2.167^ TCID_50_/0.1 mL in the IFNγ group) at 36 hpi (Figure 2C); while at 48 hpi, the virus titer in the IFNα group dropped to 10^3.125^ TCID_50_/0.1 mL and that in the IFNγ group declined to 10^2.542^ TCID_50_/0.1 mL (Figure 2C). These results indicate that both goIFNα and goIFNγ conferred the duck cells with resistance to the virus. Then, the viral gene copies were detected at 36 hpi (IFNα: 10^4.59^ copies/200 µL, IFNγ: 10^3.82^ copies/200 µL) and 48 hpi (IFNα: 10^5.37^ copies/200 µL, IFNγ: 10^5.78^ copies/200 µL) (Figure 3), when compared to the control group, a significant decrease for both IFNα (*p* < 0.001, *p* < 0.001) and IFNγ (*p* < 0.001, *p* < 0.001) were calculated. At 36 hpi, the EGFP-positive cells of the IFNα group accounted for 3.89% of the total, followed by the IFNγ group (0.79%), both of which were lower than in the control group (approximately 50%). At 48 hpi, the infected cells comprised 17.05% of the IFNα group and 5.58% of the IFNγ group, indicating that goose IFNγ protein conferred more resistance against viral infection. The synergistic treatment of both IFNα and IFNγ proteins also have the significant inhibition effect on DPV (Figure S2).

**Figure 4:**
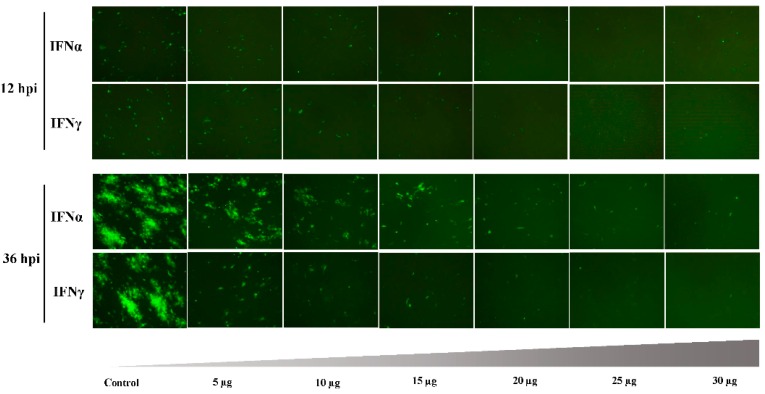
Dose-dependent inhibition of DPV replication by IFNα and IFNγ is shown by green fluorescence (Magnification 400×). To study the inhibitory effect of IFNs against DPV, DEF cells were seeded in 24-well plates at 10^5^ cells/well, treated with indicated IFNα or IFNγ, at 5, 10, 15, 20, 25 and 30 µg or left untreated (control); 12 h later, the cells were infected with DPV (10^4^ TCID_50_/well). At 12 hpi and 36 hpi, green fluorescence was examined via fluorescence microscopy, respectively (original magnification × 400).

**Figure 5:**
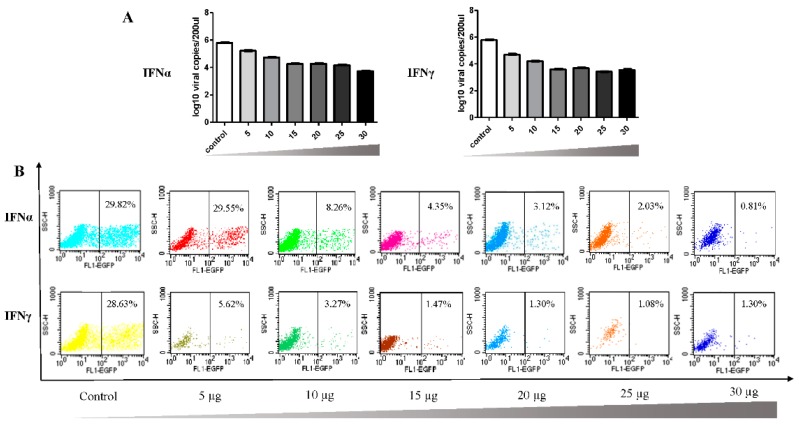
Dose-dependent inhibition of DPV replication by IFNα and IFNγ as examined by viral copy number detection (**A**) and flow cytometry analysis (**B**). Effects of different concentrations of IFNα and IFNγ on DPV DNA copy numbers. DPV UL30 DNA copies in DEF cells of control groups and experimental groups were detected by RT-qPCR at 36 hpi after protein pretreatment as above. The number of UL30 DNA copies was assessed in triplicate wells. Flow cytometry analysis of DPV-EGFP positive cells with different concentrations of IFN were also analyzed by flow cytometry. The percentage of EGFP-positive cells automatically calculated by the CellQuest software. Flow cytometry was used to analyze cells with GFP fluorescence. Plots of the data indicate the percentage of cells expressing GFP (right quadrant) above the background level of fluorescence associated with the uninfected control cells.

Then, the dose-dependent antiviral effect induced by interferons was then assessed. As shown in Figure 4, the suppression effect was not apparent at 12 hpi with increased interferon protein dose. However, at 36 hpi, the DEFs were protected by the incubation with a low dose of IFNα or IFNγ protein, and the DPV infection was efficiently controlled. As shown in Figure 5A, viral gene copy numbers were lower than in the control group after pretreatment with the indicated IFN protein. Furthermore, flow cytometry analysis showed that this inhibitory effect was dose dependent (Figure 5B), consistent with the results of the indirect fluorescence assay.

### 3.3. GEFs and DEFs Display Similar Positive Feedback Regulation by Goose IFN and Subsequent ISG Induction

**Figure 6:**
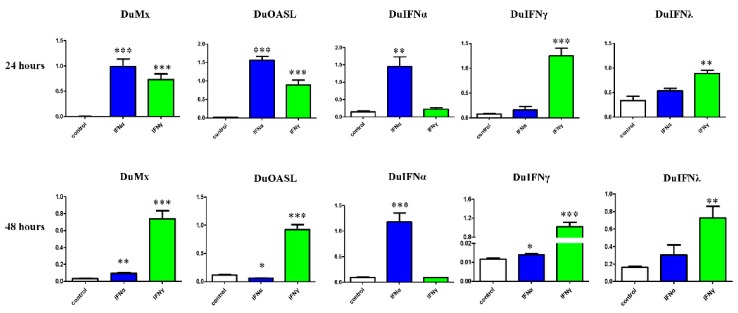
Detection of duck Mx (duMx), duck OASL (duOASL), duck IFNα (duIFNα), duck IFNγ (duIFNγ), and duck IFNλ (duIFNλ) gene expression levels in DEFs. Cells were collected at 24 h and 48 h after the goose IFNα (goIFNα) and goose IFNγ (goIFNγ) protein pretreatment (60 µg/well). The mRNA levels were measured by real time PCR and are expressed relative to β-actin mRNA. Duck embryo fibroblast (DEF) cells were seeded in 6-well plates and then pretreated with by IFNα or IFNγ protein for 12 h. Symbols show four replicate experiments. The results shown are the mean ± SEM of four samples. Significant differences compared with cells without IFN treatment are denoted by * *p* < 0.05; ** *p* < 0.01 and *** *p* < 0.001.

To understand the mechanism by which interferon inhibits DPV replication, we further investigated the expression of Mx, OASL, and three types of duck IFNs. The results showed that the selected duck ISGs (Mx and OASL), duIFNα, and duIFNγ were significantly upregulated in DEF after the pretreatment with goose IFNα and IFNγ proteins for 24 h (Figure 6). Thus, the duck IFN system in the duck embryo fibroblast cells could be highly induced by goose IFN proteins. The level of duIFNα was more than 100 times lower than that of duIFNγ (Figure 6). Significant upregulation in the expression of duMx and duOASL gene expression was observed. We also compared the expression of IFNα, IFNγ and their target genes in goose embryonic fibroblasts (GEFs) and DEFs in response to goose interferon stimulation (Figure 6 and Figure S3). In goose primary cells (Figure S3), similar patterns in ISGs and the self-feedback loop of IFN were observed for goose type I and type II IFNs.

## 4. Discussion

Interferons, which function as regulators of the antiviral immune response by interacting with cognate receptors on the surface of cells, have been intensively studied for a long time. Although interferons have been identified in avian species, the research into their antiviral activity has lagged behind research in mammals. Nonetheless, interferons have key function in the first line of defense against virus infection, bridging the innate and adaptive immune responses. As reported previously, aquatic birds (e.g., the geese) have a critical role in the transmission and dissemination of a wide range of important human and animal pathogens (e.g., avian influenza virus). Sequence and phylogenetic analyses have indicated that goose IFNα shared its highest identity with duck IFNα [9]. In addition, evolutionary and structural analyses demonstrated a remarkable degree of structural conservation for IFNγ among vertebrates during the evolution of immune genes [24]. Goose IFNα is reported to significantly reduce goose paramyxovirus (GPMV) plaques significantly in goose fibroblasts and restrict the VSV-EGFP in duck fibroblasts [9]. Goose IFNγ also induced an antiviral state against GPMV in goose fibroblasts and inhibited VSV-GEFP replication in duck fibroblasts [10]. Based on the similar genomic structure and previous studies, we suspected that goose IFNα and IFNγ may confer cross-species antiviral activity in heterologous cells.

In our study, goose IFNα and IFNγ were shown to possess an ability to inhibit DPV replication during late stage in duck cells in vitro. Conventionally, the type I interferons are the major components of the innate immune response of hosts. In the present study, the type II interferon, also known as IFNγ, was identified as possessing considerable antiviral activity upon DPV infection. Indeed, during infection with DPV-EGFP, the viral gene copies decreased markedly upon addition of goIFNα and goIFNγ. Therefore, it can be simply inferred that type I interferons induce an antiviral response and provide the protection against DPV-EGFP. Interestingly, the functional type II IFN antiviral system also appeared to be involved in this process. Both duck type I IFN and type II IFN system contribute to the host antiviral effect on DPV. Furthermore, DEF pretreatment with IFN protein resulted in a dose-dependent inhibition of DPV-EGFP compared to the control.

One possible explanation is that the interferons might modulate IFN antiviral proteins signaling transduction via expression of interferon-stimulated genes [25]. To fully understand the role of interferons in the primary immune response following interferon activation, we sought to determine the expression level of interferon-stimulated genes in cells activated by interferons. We speculated that the transcriptional levels of ISGs may contribute to the duck cellular antiviral state triggered by goose IFNα and IFNγ. ISGs encode specific proteins that have antiviral properties, interfering with viruses at different stages of their replication cycle. Although IFNα and IFNγ share no obvious sequence or structural homology, there do exhibit functional similarities, such as overlap in the ISGs they induce. Interestingly, IFNs have been reported to have strong antiviral activity in heterologous cells of different types. For example, the human interferons were less active in heterologous rabbit cells than in homologous human fibroblasts [26]. Additionally, a greater activity was observed in bovine and porcine cells treated with human interferons than that in homologous human cells [27]. Heterologous activity of mouse interferons was also reported [28], and pigeon IFNγ has been reported to be active in a chicken macrophage cell line [29].

Therefore, it appears that goose interferons induce the expression of a series of interferon-stimulated genes in duck cells, as the selected duck ISGs, including Mx and OASL, were all upregulated with the goose IFNs activation. Taken together, these results indicate that interferons can enhance an effective host immune response to protect against infection. The antiviral effect of goose IFNs in heterogeneous duck cells may be related to a higher production of ISGs, such as Mx and OASL. The Mx protein, a potent antiviral restriction factor, inhibits a diverse range of viruses at unique steps in their life cycle, including inhibition of RNA polymerase, viral nucleocapsids production, genome replication and chromosomal integration [14,30]. Both duMx and goMx can be induced by goose IFNα and IFNγ. In addition, the OAS gene family can be induced by both IFNs, then the OAS enzymes expressed participate in the synthesis of 2′-5′-linked oligoadenylates from ATP, which can subsequently stimulate the RNase L for the degradation of viral and cellular RNAs [31,32,33]. In this study, the goose OASL and duck OASL were likely induced, suggesting that duOASL may assist in this antiviral process.

During the early phase of viral infection in mammals, IFNs recognize IFNR via autocrine and paracrine pathways and induce the phosphorylation of IRF7 [34], a key regulator of the high production of IFNs in the late stage [35]. At both the early and late stages, interferons contribute to the host’s strong antiviral defense. However, in birds, some immune defense genes and regulatory cytokines have been lost during evolution [36], and self-regulation in avian has not been reported to date. Even more interesting is our observation that the goose IFN can activate the feedback loop in duck cells. The expression of three types of goose IFNs in GEFs have been examined thus far, and the positive feedback loop of type I IFN and type II IFN has been confirmed in both homologous (goose) and heterogeneous (duck) cells (Figure 6 and Figure S3). These similarities may also partly explain the self-feedback of interferons, which was also activated in two species. This type of the positive feedback provides the host cells with long-lasting and strong antiviral responses. Further investigation confirmed that the auto-amplification loop of IFNs was also involved in the defense against viral infection.

## 5. Conclusions

In conclusion, this study highlights the antiviral activity of goose interferons and their cross-species activity. These functions appear to be related to the modulation of the IFN-induced antiviral proteins, such as Mx and OASL. It is important to elucidate the detailed mechanisms of the goose antiviral immune system, as such details may provide invaluable insights for the development of animal antiviral therapeutics. Overall, studies of interferons will help in the development of prophylactic and therapeutic approaches against viral disease, and the functional significance of interferons deserves further investigation.

## Figures and Tables

**Table 1:** List of primers used in this study and their sequences.

Species	Primer Name	Nucleotide Sequence
Goose	IFNα (F)	CAGCACCACATCCACCAC
	IFNα (R)	TACTTGTTGATGCCGAGGT
	IFNγ (F)	TGAGCCAGATTGTTTCCC
	IFNγ (R)	CAGGTCCACGAGGTCTTT
	IFNλ (F)	GAGCTCTCGGTGCCCGACC
	IFNλ (R)	CTCAGCGGCCACGCAGCCT
	Mx (F)	TTCACAGCAATGGAAAGGGA
	Mx (R)	ATTAGTGTCGGGTCTGGGA
	OASL (F)	CAGCGTGTGGTGGTTCTC
	OASL (R)	AACCAGACGATGACATACAC
	actin (F)	CCGTGACATCAAGGAGAA
	actin (R)	GAAGGATGGCTGGAAGAG
Duck	IFNα (F)	TCCTCCAACACCTCTTCGAC
	IFNα (R)	GGGCTGTAGGTGTGGTTCTG
	IFNγ (F)	CATACTGAGCCAGATTGTTACCC
	IFNγ (R)	TCACAGCCTTGCGTTGGA
	IFNλ (F)	GTGCCTGACCGACTCCTCCT
	IFNλ (R)	CCCAGAGGGCTGATGCGAAG
	Mx (F)	TGCTGTCCTTCATGACTTCG
	Mx (R)	GCTTTGCTGAGCCGATTAAC
	OASL (F)	TCTTCCTCAGCTGCTTCTCC
	OASL (R)	ACTTCGATGGACTCGCTGTT
	β-actin (F)	GATCACAGCCCTGGCACC
	β-actin (R)	CGGATTCATCATACTCCTGCTT
DPV	UL30 (F)	TTTCCTCCTCCTCGCTGAGTG
	UL30 (R)	CCAGAAACATACTGTGAGAGTG
Plasmid construction	pcDNA-IFNα (F)	CTA GCTAGC GACATGGAC TGCAGCCCCCTGCGCCTCCACGACAG
	pcDNA-IFNα (R)	CGG GAATTC TTA GTGGTGGTGGTGGTGGTG GCGCATGGCGCGGGTGAGGCG
	pcDNA-IFNγ (F)	CTA GCTAGC GACATGGAC TGTTCTGGAAGTGCTCTATTTCTTAG
	pcDNA-IFNγ (R)	CGG GAATTC TTA GTGGTGGTGGTGGTGGTG ACATCTGCATCTCTTTGGAGAC
Plasmid construction	pEGFP-IFNα (F)	ATCTCGAGCTCAAGCTTC GAATTC ATGCCTGGGCCATCAGCCCCAC
(one-step cloning)	pEGFP-IFNα (R)	GGTGGATCCCGGGCCCGC GGTACC AC GCGCATGGCGCGGGTGAGGCG
	pEGFP-IFNγ (F)	ATCTCGAGCTCAAGCTTC GAATTC GCCACC ATGACTTGCCAGACCTACTGCTTG
	pEGFP-IFNγ (R)	GGTGGATCCCGGGCCCGC GGTACC AC ACATCTGCATCTCTTTGGAGAC

## Supplementary Materials

Supplementary File 1

## Abbreviations

The following abbreviations are used in this manuscript:

PRRs	pattern recognition receptors
PAMPs	pathogen-associated molecular patterns
IFN	interferon
Mx	myxovirus resistance
OASL	oligoadenylate synthetases-like
ISGs	IFN-stimulated genes
DPV	duck plague virus
IBV	infectious bronchitis virus
IBDV	infectious bursal disease virus
MDV	Marek’s disease virus
