# Supplementary material for: Cross-Species Antiviral Activity of Goose Interferons against Duck Plague Virus Is Related to Its Positive Self-Feedback Regulation and Subsequent Interferon Stimulated Genes Induction
Source: Viruses. 2016 Jul 18;8(7):195. doi: 10.3390/v8070195 (PMC4974530; doi:10.3390/v8070195)
Supplement: Supplementary File 1 [file viruses-08-00195-s001.pdf]

Supplementary Materials: Cross-Species Antiviral Activity of Goose Interferons against Duck Plague Virus Is Related to Its Positive Self-Feedback Regulation and Subsequent Interferon Stimulated Genes Induction

Hao Zhou, Shun Chen, Qin Zhou, Yunan Wei, Mingshu Wang, Renyong Jia, Dekang Zhu, Mafeng Liu, Fei Liu, Qiao Yang, Ying Wu, Kunfeng Sun, Xiaoyue Chen and Anchun Cheng

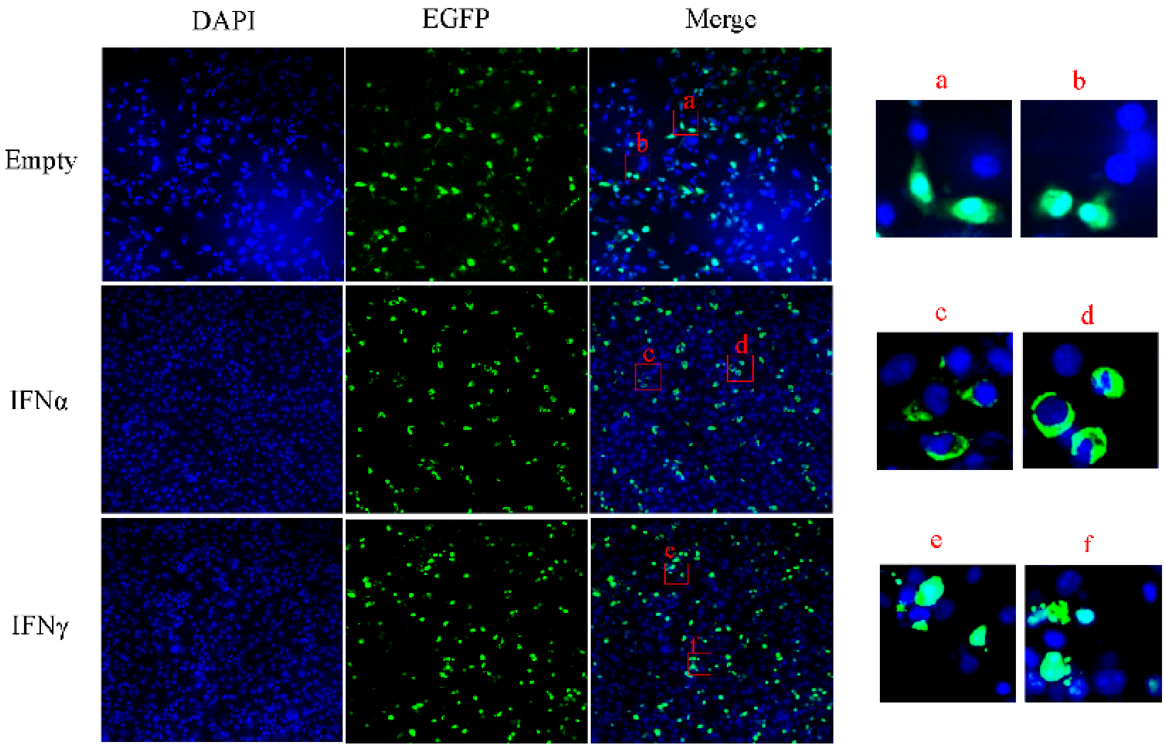

Figure S1. Subcellular localization of goose IFNα and IFNγ protein in BHK21 cells. BHK21 cells plated onto glass coverslips and incubated overnight in 6-well plates were transfected with 2 µg pEGFP-IFNα or pEGFP-IFNγ. At 24 h post transfection, the cells were fixed for immunofluorescence analysis (original magnification × 400). The nuclei were stained with DAPI (blue). Higher magnification views of the area indicated by the box are shown on the right.

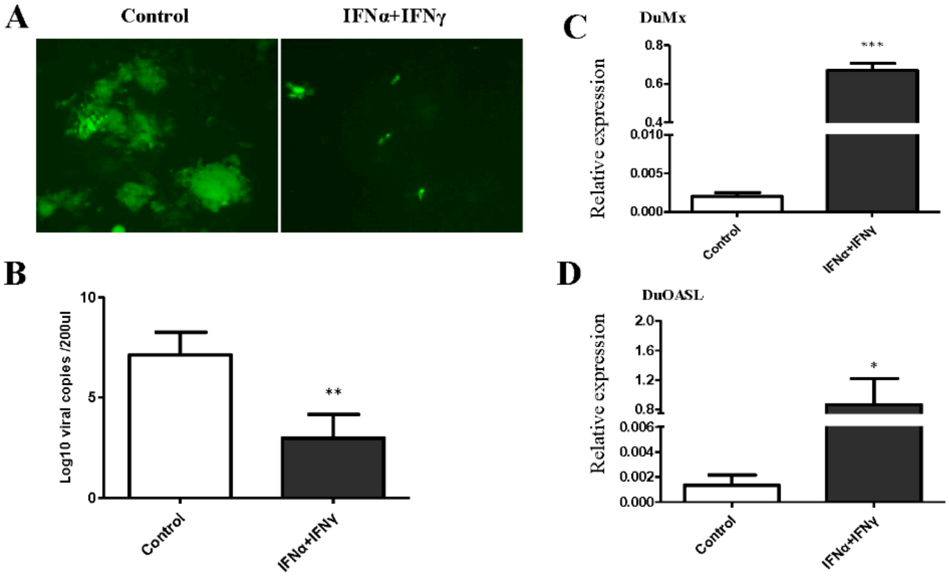

Figure S2. The synergistic effect of both goose IFNα and IFNγ proteins on DPV replication. (**A**) Inhibition of DPV replication by both goose IFNα and IFNγ proteins is shown by green fluorescence (Magnification 400×). DEF cells were seeded in 6-well plates, 12 h later, treated with indicated both IFNα and IFNγ, at respective 60 µg per well or left control; 12 h later, the cells were then infected by DPV-EGFP (4 × 104 TCID$_{50}$/well). At 36 hpi, green fluorescence was examined via fluorescence microscopy; (**B**) The effect of co-treatment of both IFNα and IFNγ protein on the viral copies of DPV (** $p < 0.01$); (**C**,**D**) The levels of DuMx and DuOASL gene expression in GEFs after treatment with the co-treatment of both goIFNα and goIFNγ proteins were detected by qRT-PCR. The data represent the mean ± SEM per group ($n = 3$). Significant differences compared with cells without goIFNα or goIFNγ treatment are denoted by (* $p < 0.05$) and (*** $p < 0.001$).

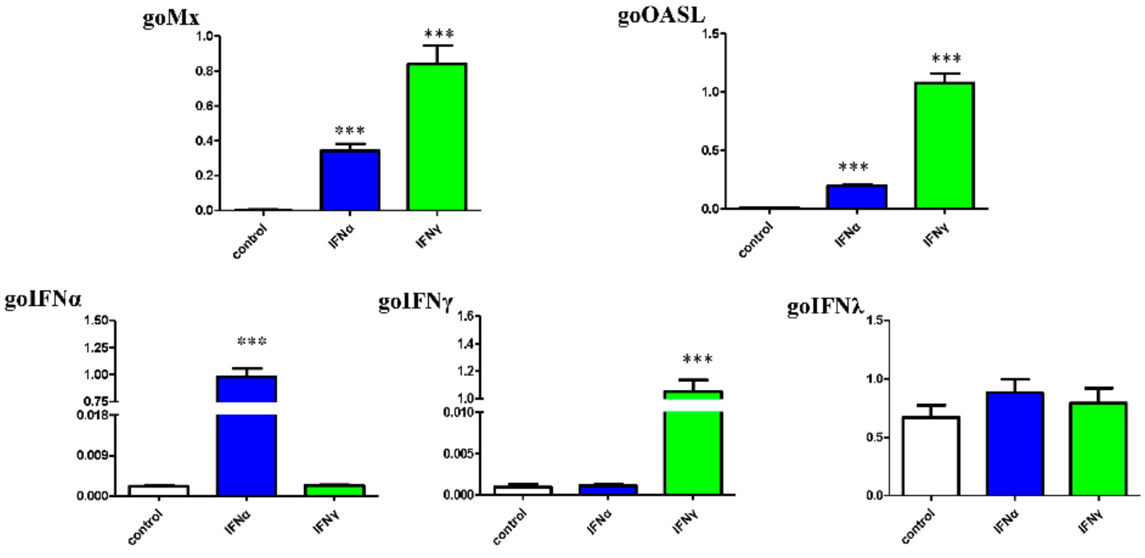

Figure S3. Effect of goose interferons on goose IFNs and ISGs in GEFs. The levels of goMx, goOASL, goIFNα, goIFNγ, and goIFN λ gene expression in GEFs after treatment with the goIFNα and goIFNγ proteins (60 µg/well) were detected by qRT-PCR. Total RNA was extracted from GEF cells after incubation with interferon for 24 h. The internal control was β-actin. The data represent the mean ± SEM from four replicates per group. Significant differences compared with cells without treatment goIFNα or goIFNγ treatment are denoted by ** $p < 0.01$.

© 2016 by the authors. Submitted for possible open access publication under the terms and conditions of the Creative Commons Attribution (CC-BY) license (http://creativecommons.org/licenses/by/4.0/).
